# First-Time Fathers’ Knowledge and Attitudes Toward Breast Milk and Breastfeeding: A Cross-Sectional Study

**DOI:** 10.3390/healthcare14213803

**Published:** 2026-11-06

**Authors:** Aslı Ural

**Affiliations:** Faculty of Health Sciences, Yozgat Bozok University, Yozgat 66900, Türkiye; asli.ural@yobu.edu.tr

**Keywords:** breast milk, breastfeeding, first-time fathers, paternal support, breastfeeding knowledge, breastfeeding attitudes, good health and well-being

## Abstract

**Highlights:**

**What are the main findings?**

- First-time fathers generally had positive attitudes toward breast milk and breastfeeding but showed knowledge gaps regarding colostrum, infant formula, and their role in breastfeeding decisions. Only 4.8% correctly answered the item assessing knowledge of this role.
- Fathers who had received antenatal education had higher breastfeeding knowledge scores (*p* = 0.025), but antenatal education was not significantly associated with knowledge scores after multivariable adjustment (*p* = 0.110).

**What are the implications of the main findings?**

- Education on breast milk and breastfeeding should address fathers’ knowledge gaps, particularly their role in breastfeeding decisions and partner support.
- Including first-time fathers in antenatal and postpartum education on breast milk and breastfeeding may strengthen family-centered breastfeeding support.

**Abstract:**

Objective: This study evaluated first-time fathers’ knowledge and attitudes toward breast milk and breastfeeding. Methods: This descriptive, cross-sectional study included 289 first-time fathers at a hospital in Yozgat, Türkiye, between January and June 2026. Data were collected using an information form, a 15-item Breast Milk and Breastfeeding Knowledge Form, and a five-item Breast Milk and Breastfeeding Attitude Form. Data were analyzed using descriptive statistics, Mann–Whitney U and Kruskal–Wallis tests, and multivariable robust regression. Results: Of the fathers, 31.8% had received antenatal education on pregnancy, childbirth, and the postpartum period, while 34.9% had received postpartum education on breast milk and breastfeeding. The median number of correct responses was 10 out of 15. Correct response rates were highest for exclusive breastfeeding during the first six months (98.6%) and lowest for fathers’ involvement in breastfeeding decisions (4.8%), colostrum (11.1%), and infant formula (11.4%). Regarding attitudes, 94.5% rated early breastfeeding initiation, 98.3% rated breast milk for infant growth and development, and 93.4% rated partner support as very important. Fathers who had received antenatal education on pregnancy, childbirth, and the postpartum period had significantly higher knowledge scores than those who did not receive such education (*p* = 0.025). However, antenatal education was not significantly associated with knowledge scores after multivariable adjustment (*p* = 0.110), and the robust regression model was not statistically significant (F = 1.042, *p* = 0.412; R^2^ = 0.047). Conclusions: First-time fathers expressed positive breastfeeding attitudes despite important knowledge gaps. Addressing these gaps in antenatal and postpartum education may support a more comprehensive approach to family-centered breastfeeding care.

## 1. Introduction

The initiation and continuation of breastfeeding are associated not only with mothers’ knowledge, attitudes, and motivation but also with the support they receive from their families and social environment [1,2]. Within this support network, fathers play an important role in decisions about breastfeeding and its continuation [3,4,5,6]. Through emotional support, practical assistance, and shared caregiving responsibilities, fathers may strengthen mothers’ breastfeeding self-efficacy and motivation, thereby positively influencing breastfeeding outcomes [7]. Consistent with this mechanism, fathers’ self-efficacy in providing breastfeeding support has been associated with exclusive breastfeeding outcomes [6,8], while their self-efficacy in newborn care has been positively associated with attitudes toward breastfeeding and involvement in the breastfeeding process [9].

Fathers’ ability to provide effective breastfeeding support is shaped by their attitudes, knowledge, self-efficacy, and the broader context in which support is provided. Positive attitudes toward breastfeeding have been associated with breastfeeding initiation, continuation, and fathers’ involvement in the breastfeeding process [10,11,12,13], while knowledge of the benefits of breastfeeding for mothers and infants and of recommended breastfeeding practices has been reported to support fathers in providing more effective assistance to their partners [12,14]. However, fathers’ knowledge levels vary, and their involvement is also shaped by personal, cultural, religious, social, and environmental factors, as well as employment and working conditions [15]. Family support and access to healthcare services further influence fathers’ participation in breastfeeding support [16]. Even when fathers are willing to support breastfeeding, uncertainty about how to assist their partners may lead to feelings of inadequacy, exclusion, or helplessness [17,18]. Low self-efficacy and limited practical skills may further restrict their active participation. In a qualitative study involving fathers whose partners had recently given birth, low self-efficacy constrained practical involvement, while difficulties with positioning, latching, and problem-solving contributed to feelings of inadequacy during breastfeeding [16].

These findings suggest that positive attitudes alone are insufficient for effective paternal involvement in breastfeeding and that adequate knowledge and support skills are also required. Paternal breastfeeding support is considered a multidimensional construct comprising emotional, informational, and practical dimensions [19]. Including fathers in breastfeeding education and support interventions has been reported to have positive effects on breastfeeding initiation, continuation, and exclusive breastfeeding outcomes [1,20,21,22]. In addition, a recent systematic review and meta-analysis showed that family-based breastfeeding interventions not only increased exclusive breastfeeding rates but also improved breastfeeding knowledge, partner and family support, and father–infant attachment [23]. Nevertheless, breastfeeding education and interventions have predominantly focused on mothers, while fathers’ needs and roles in the breastfeeding process have received less attention [15,24]. Fathers have reported feeling overlooked in breastfeeding education, perceiving available services as primarily mother-focused, and feeling excluded from decision-making processes [15,17]. They have also identified a need for information on how to manage breastfeeding difficulties, how to become involved in the care of a breastfed infant, and how best to support their breastfeeding partners [25].

First-time fathers represent a group that warrants particular attention because they have no previous experience of parenthood or providing breastfeeding support. During this period, fathers are adapting to new parental responsibilities while also learning how to support their partners throughout the breastfeeding process [10,26]. Limited knowledge and low self-efficacy may hinder this group’s active involvement in breastfeeding support [8,16]. Studies conducted in Türkiye have also shown that fathers are generally willing to support breastfeeding. However, their knowledge of breast milk and breastfeeding may be limited, indicating a need for father-specific education [27,28].

Considering the existing literature, it is important to assess both knowledge and attitudes regarding breast milk and breastfeeding among first-time fathers. However, evidence on first-time fathers’ knowledge and attitudes, particularly the factors associated with their breastfeeding knowledge, remains limited. This study aimed to evaluate first-time fathers’ knowledge and attitudes regarding breast milk and breastfeeding, identify gaps in their knowledge, and examine the potential associations between breastfeeding knowledge and sociodemographic, obstetric, and education-related characteristics. The findings may contribute to the development of father-focused education and family-centered breastfeeding support programs.

The study sought to answer the following research questions:

What are first-time fathers’ levels of knowledge and attitudes regarding breast milk and breastfeeding?

Do breastfeeding knowledge scores differ according to first-time fathers’ sociodemographic and obstetric characteristics?

Which sociodemographic, obstetric, and education-related characteristics are associated with first-time fathers’ total breastfeeding knowledge scores after adjusting for other variables?

## 2. Materials and Methods

### 2.1. Research Design and Participants

This descriptive, cross-sectional study was conducted in the Department of Obstetrics and Gynecology of a public hospital in Yozgat, Türkiye, between 1 January and 30 June 2026. The study was conducted in accordance with the Strengthening the Reporting of Observational Studies in Epidemiology (STROBE) guidelines [29]. Participants were first-time fathers whose partners were hospitalized in the postpartum ward during the study period. No a priori sample size calculation was performed. During the data collection period, all first-time fathers who met the eligibility criteria were approached. Of the 310 eligible fathers approached, 14 declined participation, and seven were excluded from the final analysis due to incomplete questionnaires. Consequently, 289 fathers were included in the final sample. Men were eligible if their partners were hospitalized in the postpartum ward and their newborns were rooming-in with their mothers; they were aged ≥18 years; they were first-time fathers with no previous living children; they were able to read and write in Turkish; and they agreed to participate in the study. Fathers who self-reported a mental health condition that could interfere with their ability to understand or complete the questionnaire were excluded from the study.

### 2.2. Measurements

Data were collected using a three-part data collection form developed by the researcher.

#### 2.2.1. Descriptive Information Form

The Descriptive Information Form was developed by the researcher based on the relevant literature. The form consisted of 15 questions assessing participants’ sociodemographic characteristics and selected characteristics related to their partners’ pregnancy and childbirth [14,15,16,28].

#### 2.2.2. Breastfeeding and Breast Milk Knowledge Form

The Breastfeeding and Breast Milk Knowledge Form was developed by the researcher to assess first-time fathers’ knowledge of breast milk and breastfeeding. The items were generated based on posters and brochures on breast milk and breastfeeding published by the General Directorate of Public Health of the Ministry of Health of the Republic of Türkiye, as well as the Ten Steps to Successful Breastfeeding. The initial item pool consisted of 30 items [30].

The draft form was evaluated by five experts comprising two academics specializing in women’s health and disease nursing, one midwife, one neonatal nurse, and one lactation consultant. The experts assessed the items in terms of content relevance, clarity, and comprehensibility. Content validity was evaluated using the Davis [31] method. Each item was rated on a four-point scale (1 = not relevant, 2 = somewhat relevant, 3 = quite relevant, and 4 = highly relevant). For the analysis, ratings of 3 or 4 were coded as 1 (relevant), whereas ratings of 1 or 2 were coded as 0 (not relevant). The item-level content validity index (I-CVI) was calculated as the proportion of experts rating each item as relevant, and the scale-level content validity index based on the average method (S-CVI/Ave) was calculated as the mean of the I-CVI values. An I-CVI threshold of 0.80 was used for item selection [32].

The initial 30-item pool had an S-CVI/Ave of 0.473. Of the 30 items, 15 had I-CVI values ranging from 0.80 to 1.00 and were retained, whereas the remaining 15 items had I-CVI values below the 0.80 threshold and were excluded. Thus, the final Knowledge Form consisted of 15 items. The internal consistency of the final 15-item Knowledge Form was assessed using the Kuder–Richardson Formula 20 (KR-20), yielding a coefficient of 0.586.

Each item was answered as “True,” “False,” or “I don’t know.” Correct responses were scored as 1, whereas incorrect and “I don’t know” responses were scored as 0. “True” was scored as correct for Items 1, 2, 5–9, 11, 12, 14, and 15, whereas “False” was scored as correct for Items 3, 4, 10, and 13. The total knowledge score ranged from 0 to 15, with higher scores indicating greater knowledge of breast milk and breastfeeding. No cut-off score was established, and participants were not categorized according to their knowledge scores.

#### 2.2.3. Breastfeeding and Breast Milk Attitude Form

The Breastfeeding and Breast Milk Attitude Form was developed by the researcher to assess first-time fathers’ attitudes toward breastfeeding. The items were generated based on posters and brochures on breast milk and breastfeeding published by the General Directorate of Public Health of the Ministry of Health of the Republic of Türkiye, as well as the Ten Steps to Successful Breastfeeding [30]. The form comprised five items addressing attitudes toward the early initiation of breastfeeding, the role of breast milk in infant growth and development, the benefits of breastfeeding for maternal health, paternal support during breastfeeding, and the importance of antenatal education on breast milk and breastfeeding.

The items were evaluated by the same five experts for content relevance, clarity, and comprehensibility. Based on the experts’ feedback, no modifications to the items were considered necessary. Each item was rated on a five-point Likert-type response format with the following categories: “not important at all,” “not important,” “slightly important,” “moderately important,” and “very important.” The five items were designed to assess distinct breastfeeding-related attitudes and were therefore analyzed individually rather than as a single scale. As the validity of a total attitude score was not established, no total score was calculated.

No pilot testing was performed for either form.

### 2.3. Data Collection Process

Data were collected through face-to-face interviews in the Department of Obstetrics and Gynecology between 1 January and 30 June 2026. Participants who met the eligibility criteria were informed by the researcher about the study, including its purpose, scope, and voluntary nature. Written and verbal informed consent were obtained from all fathers who agreed to participate before data collection. Participants completed the data collection form under the researcher’s supervision, and completion required approximately 10–15 min.

### 2.4. Statistical Analysis

Statistical analyses were performed using R software (version 4.5.0) and NCSS software (version 11). The normality of continuous variables was assessed using the Shapiro–Wilk and Kolmogorov–Smirnov tests. The Mann–Whitney U test was used to compare non-normally distributed continuous variables between two independent groups, whereas the Kruskal–Wallis H test was used for comparisons among three or more independent groups. Associations between non-normally distributed continuous variables were examined using Spearman’s rho correlation analysis.

Multivariable robust regression analysis was performed using the MASS package in R to examine factors associated with breastfeeding knowledge scores while accounting for potential confounding variables. Covariates were selected based on previous literature and their potential associations with breastfeeding knowledge.

The content validity of the researcher-developed Breastfeeding and Breast Milk Knowledge Form was assessed using the Davis [31] method. The item-level content validity index (I-CVI) and the scale-level content validity index based on the average method (S-CVI/Ave) were calculated. An I-CVI threshold of 0.80 was used for item selection [32].

Continuous variables were reported as mean ± standard deviation (SD) and median (minimum–maximum), while categorical variables were presented as frequency (percentage). A two-sided *p*-value < 0.05 was considered statistically significant.

### 2.5. Ethical Considerations

The study was approved by the Social and Human Sciences Ethics Committee of Yozgat Bozok University (approval date: 17 December 2025; Decision No. 31/44), and institutional permission was obtained before the commencement of the study. The study was conducted in accordance with the principles of the Declaration of Helsinki. Written and verbal informed consent were obtained from all participants before data collection. Participation was voluntary, and the confidentiality of participants’ information was ensured.

## 3. Results

The participants had a mean age of 28.74 ± 4.72 years, while the mean gestational age of their partners was 38.42 ± 2.59 weeks. Overall, 50.2% of the participants had a middle school education, 68.5% were employed as workers, 54.3% reported that their income was equivalent to their household expenses, and 66.8% lived in nuclear families. The pregnancy was planned in 90.3% of participants’ partners, 58.1% of their partners delivered by cesarean section, and 51.3% of the infants were male. Overall, 31.8% of fathers had received antenatal education on pregnancy, childbirth, and the postpartum period, while 34.9% had received postpartum education on breast milk and breastfeeding. Nearly all participants (98.3%) reported that their infant needed breast milk (Table 1).

The analysis of participants’ responses to the statements regarding breastfeeding and breast milk showed that the proportion of correct responses ranged from 4.8% to 98.6%. The highest proportions of correct responses were observed for the statements that infants should be exclusively breastfed for the first six months and that breastfeeding should continue until two years of age and beyond (98.6%), that every infant has the right to receive breast milk (98.3%), and that breast milk supports the infant’s immune system (96.2%). The lowest proportions of correct responses were observed for the statements that a mother should not breastfeed her infant if the father does not want her to (4.8%), that the first milk (colostrum) is not beneficial to the infant (11.1%), that infant formula is more nutritious than breast milk (11.4%), and that breastfeeding negatively affects breast appearance (22.1%). A median of ten breastfeeding-related knowledge statements were answered correctly (Table 2).

When participants’ responses to the breastfeeding and breast milk attitude items were examined, most considered initiation of breastfeeding immediately after birth (94.5%), the importance of breast milk for infant growth and development (98.3%), and partner support for breastfeeding (93.4%) to be “very important.” Overall, 61.9% of the participants considered the beneficial effects of breastfeeding on maternal health to be “very important,” while 73.0% of the participants considered prenatal education on breast milk and breastfeeding to be “very important” for successful breastfeeding (Table 3).

When participants’ numbers of correct responses regarding breastfeeding and breast milk were compared according to their sociodemographic and obstetric characteristics, no significant differences were observed according to educational attainment, employment status, income level, pregnancy intendedness, family structure, mode of delivery, or infant sex (*p* > 0.05). Participants who had received education on pregnancy, childbirth, and the postpartum period during the antenatal period had significantly higher numbers of correct responses than those who had not received such education (*p* = 0.025), with a small effect size (d = 0.265, 95% CI: 0.017–0.514). In contrast, the number of correct responses did not differ significantly according to whether participants had received education on breast milk and breastfeeding during the postpartum period (*p* = 0.061) (Table 4).

To further examine factors associated with breastfeeding knowledge scores, a multivariable robust regression analysis was performed. The overall model was not statistically significant (F = 1.042, *p* = 0.412; R^2^ = 0.0469). Fathers who had received antenatal education on pregnancy, childbirth, and the postpartum period had significantly higher breastfeeding knowledge scores than those who had not received such education in the unadjusted analysis (*p* = 0.025). However, antenatal education was not significantly associated with breastfeeding knowledge scores after adjustment for other variables (*p* = 0.110). The detailed regression results are presented in Supplementary Table S1.

No significant correlation was found between participants’ age and the number of correct responses regarding breastfeeding and breast milk (r = 0.013; *p* = 0.831). Similarly, no significant correlation was observed between their partners’ gestational age and the number of correct responses (r = 0.023; *p* = 0.702) (Table 5).

## 4. Discussion

The fact that half of the participants had completed primary education (50.2%) was a notable characteristic of our cohort in terms of educational level. In addition, 68.2% of the participants had not received prenatal education on pregnancy, childbirth, and the postpartum period, while 65.1% had not received education on breast milk and breastfeeding. These findings indicate that fathers’ educational needs regarding breastfeeding are not being adequately addressed. In the study by Taşpınar et al. [28], although fathers had a positive attitude toward breastfeeding, their knowledge of lactation and breastfeeding was found to be limited, and 48.8% of participants reported that they would like to participate in a breastfeeding education program specifically designed for fathers. These findings support the importance of including fathers in breastfeeding education beginning in the prenatal period. In contrast, the finding that 98.3% of participants stated that infants need breast milk indicates that they held a positive view of the importance of breastfeeding. However, this positive attitude may not always be accompanied by an adequate level of knowledge. Similarly, in the study by Taşpınar et al. [28], although 92.1% of fathers stated that they wanted their infants to be breastfed, only 58.6% had discussed the issue with their partners, and their knowledge regarding breastfeeding was reported to be limited. The relationship between fathers’ knowledge and attitudes and breastfeeding outcomes has also been demonstrated in several studies. Positive paternal attitudes have been reported to be associated with a greater likelihood of breastfeeding [10]. In addition, fathers’ knowledge and attitude scores regarding breastfeeding have been shown to be associated with exclusive breastfeeding at hospital discharge [14]. Hergholi et al. [7] reported that paternal self-efficacy was positively associated with maternal empowerment during the breastfeeding process. Taken together, these findings, along with the high proportion of fathers in our study who had not received education, underscore the importance of more effectively integrating fathers into structured breastfeeding education beginning in the prenatal period.

In the present study, the proportions of correct responses to basic knowledge statements regarding exclusive breastfeeding during the first six months (98.6%), every infant’s right to receive breast milk (98.3%), and the role of breast milk in supporting the infant’s immune system (96.2%) were remarkably high. Fathers have been reported to generally possess knowledge of the benefits of breast milk and basic breastfeeding principles, although their level of knowledge may vary [15]. In contrast, the proportions of correct responses to the misconceptions that colostrum is not beneficial for the infant (11.1%) and that infant formula is more nutritious than breast milk (11.4%) were quite low. Misconceptions that colostrum is inadequate or of little value and that infant formula may be more nutritious have been reported among fathers [15]. The fact that only 22.1% of participants correctly responded to the statement that breastfeeding negatively affects breast appearance also indicates the persistence of certain misconceptions regarding breastfeeding. The most striking finding was that only 4.8% of participants correctly responded to the statement, “If the father does not want breastfeeding, the mother should not breastfeed the infant.” This finding may suggest a knowledge gap regarding the father’s role in breastfeeding decisions. However, responses to this item may also reflect normative beliefs, gender roles, and sociocultural attitudes rather than breastfeeding knowledge alone. Fathers’ involvement in breastfeeding decisions and the breastfeeding process has been shown to be important for breastfeeding outcomes [6]. The need to educate fathers about breastfeeding and include them in prenatal breastfeeding education has also been emphasized previously [28]. Similarly, Gebremariam et al. [25] found that fathers considered learning how to support their partners, manage breastfeeding challenges, and become involved in the breastfeeding process particularly important. Taken together, these findings suggest that father-focused education should address knowledge gaps regarding colostrum, infant formula, and the effects of breastfeeding on maternal health, while also addressing parental roles in breastfeeding decisions.

Participants’ attitudes toward breastfeeding and breast milk were generally positive. Most fathers rated breastfeeding initiation immediately after birth (94.5%), the importance of breast milk for infant growth and development (98.3%), and partner support for breastfeeding (93.4%) as “very important.” These findings were consistent with previous studies reporting that fathers may have positive attitudes toward the benefits of breastfeeding, particularly for infants [14]. The high importance participants placed on partner support was also noteworthy. Paternal support has been reported to have positive effects on breastfeeding initiation and continuation [2]. A qualitative study conducted among African American fathers similarly reported that fathers generally had positive attitudes toward breastfeeding but felt that they needed better preparation for their roles in supporting their partners [33]. In contrast, 61.9% of participants rated the importance of breastfeeding for maternal health as “very important,” whereas 30.5% rated it as “not important at all.” This polarization in responses may reflect differences in fathers’ knowledge of the maternal health benefits of breastfeeding, as well as their personal beliefs and sociocultural attitudes. These findings suggest that the maternal benefits of breastfeeding should be more explicitly addressed in father-focused breastfeeding education. Lacerda et al. [34] reported that adolescent fathers were aware of the benefits of breastfeeding for child health but did not mention its benefits for mothers, families, or society. It has also been previously reported that fathers may evaluate breastfeeding primarily from an infant-centered perspective and may have knowledge gaps regarding its benefits for mothers [15]. A positive finding was that 73.0% of participants considered prenatal breastfeeding education to be “very important,” suggesting that fathers may be receptive to being included in the educational process. Given that breastfeeding education involving fathers may contribute to improved breastfeeding outcomes [1], these findings suggest the importance of including fathers in structured prenatal breastfeeding education programs. Similarly, a systematic review and meta-analysis of 19 studies found that family-centered breastfeeding interventions involving key family members (e.g., fathers and grandparents) were associated with higher exclusive breastfeeding rates at six months postpartum, particularly when interventions included multiple components and extended across both prenatal and postnatal periods [23]. From a healthcare practice perspective, Qin et al. [35] emphasized the importance of multidisciplinary breastfeeding education for fathers, simulation-based training to develop practical skills, and continued support after hospital discharge. In a study conducted in Türkiye, Bayrak Aykan and Kırcı Temiz [13] found that fathers’ breastfeeding attitudes were positively associated with their participation in the breastfeeding process, although overall participation levels were low. This finding further emphasizes the importance of considering fathers’ actual involvement alongside their attitudes toward breastfeeding.

In this study, fathers who had received antenatal education on pregnancy, childbirth, and the postpartum period had significantly higher numbers of correct responses than those who had not received such education in the unadjusted analysis, although the effect size was small. However, antenatal education was not significantly associated with breastfeeding knowledge scores after adjustment for other variables in the multivariable robust regression analysis. Similarly, no statistically significant difference in the number of correct responses was observed between fathers who had received postpartum education on breast milk and breastfeeding and those who had not. Differences in the content and delivery of educational programs may be relevant when interpreting these findings, although these aspects were not directly assessed in the present study. Hergholi et al. [7] highlighted the importance of practical, interactive, and sustained educational approaches in supporting fathers’ knowledge, empowerment, and confidence in breastfeeding-related practices. Similarly, Taşpınar et al. [28] attributed fathers’ inadequate knowledge of breastfeeding to their insufficient inclusion in prenatal education programs and the quality of the education provided. They also reported that fathers’ educational level, family type, and previous breastfeeding/lactation education were significantly associated with their knowledge and attitudes. Sağlam et al. [36] demonstrated that fathers’ attitudes toward breastfeeding were associated with various sociodemographic and obstetric characteristics, including educational level, family type, partner’s employment status, number of children, mode of delivery, and having received information about breastfeeding. In contrast, no statistically significant differences in breastfeeding knowledge scores according to the sociodemographic or obstetric characteristics examined were identified in the present study. These differences between studies may be related to variations in sample characteristics, measurement instruments, and sociocultural contexts. Given the cross-sectional design, the observed associations should not be interpreted as evidence of causal relationships.

In the present study, neither paternal age nor partner’s gestational age was significantly associated with breastfeeding knowledge scores. Similarly, Crippa et al. [14] found no statistically significant difference in the combined breastfeeding knowledge and attitude scores according to paternal age. These findings were consistent with the multivariable robust regression analysis conducted in the present study, in which neither paternal age nor partner’s gestational age showed a statistically significant association with breastfeeding knowledge scores.

### Limitations

This study has several limitations. The single-center, cross-sectional design and the absence of an a priori sample size calculation may limit the generalizability and statistical power of the findings. Potential selection bias and social desirability bias associated with self-reported, face-to-face data collection should also be considered. Although the researcher-developed instruments underwent expert review, no pilot testing or comprehensive psychometric validation was conducted, and the Knowledge Form showed limited internal consistency (KR-20 = 0.586). Therefore, total knowledge scores should be interpreted with caution. Additionally, the item concerning paternal authority over breastfeeding decisions may reflect attitudes and gender-related beliefs rather than factual knowledge. Multiple comparisons without adjustment may have increased the risk of Type I error. Furthermore, fathers’ actual breastfeeding support behaviors and breastfeeding outcomes were not assessed. Therefore, the findings should be interpreted cautiously, and causal relationships cannot be established.

## 5. Conclusions

This study found that first-time fathers generally had positive attitudes toward breast milk and breastfeeding but had important knowledge gaps regarding colostrum, infant formula, the benefits of breastfeeding for maternal health, and fathers’ involvement in breastfeeding decisions. Fathers who had received antenatal education on pregnancy, childbirth, and the postpartum period had significantly higher breastfeeding knowledge scores than those who had not received such education in the unadjusted analysis.

These findings highlight the importance of addressing fathers’ specific knowledge gaps and clarifying their supportive roles in breastfeeding. Antenatal and postpartum breastfeeding education should consider fathers’ informational needs and encourage their involvement in breastfeeding-related discussions and support. Addressing fathers’ identified knowledge gaps in antenatal and postpartum breastfeeding education may provide a more comprehensive approach to family-centered breastfeeding support.

## Figures and Tables

**Table 1:** Descriptive characteristics of the participants (*n* = 289).

Characteristics	Mean ± SD	Med (Min–Max)
Age	28.74 ± 4.72	28 (18–44)
Gestational age of partner (weeks)	38.42 ± 2.59	39 (25–42)
	**Frequency**	**Percentage**
Educational attainment		
Primary school	5	1.7
Middle school	145	50.2
High school	91	31.5
University	48	16.6
Employment status		
Manual worker	198	68.5
Public-sector employee	19	6.6
Self-employed	65	22.5
Unemployed	7	2.4
Income level		
Income lower than expenses	88	30.5
Income equal to expenses	157	54.3
Income higher than expenses	44	15.2
Family structure		
Nuclear family	193	66.8
Extended family	96	33.2
Pregnancy intendedness		
Unplanned	28	9.7
Planned	261	90.3
Mode of delivery		
Cesarean delivery	168	58.1
Vaginal delivery	121	41.9
Infant sex		
Female	141	48.7
Male	148	51.3
Received antenatal education on pregnancy, childbirth, and the postpartum period		
Yes	92	31.8
No	197	68.2
Received postpartum education on breast milk and breastfeeding		
Yes	101	34.9
No	188	65.1
Do you think your baby needs breast milk?		
Yes	284	98.3
No	5	1.7

SD: standard deviation; Med: median; Min: minimum; Max: maximum.

**Table 2:** Distribution of participants’ responses to breast milk and breastfeeding knowledge items.

	Frequency	Percentage	Correct Response (%)
1. Infants should be exclusively breastfed for the first six months, and breastfeeding should continue until two years of age and beyond.			
True	285	98.6	98.6
I don’t know	4	1.4	
2. Breast milk is more economical than infant formula.			
True	249	86.2	86.2
False	25	8.6	
I don’t know	15	5.2	
3. The first milk produced by the breast (colostrum) is not beneficial to the infant.			
True	208	72	11.1
False	32	11.1	
I don’t know	49	17	
4. Infant formula is more nutritious than breast milk.			
True	238	82.4	11.4
False	33	11.4	
I don’t know	18	6.2	
5. Breast milk is a safe and hygienic source of nutrition for infants.			
True	255	88.2	88.2
False	8	2.8	
I don’t know	26	9	
6. Breastfeeding may protect mothers against breast and ovarian cancer later in life.			
True	220	76.1	76.1
False	4	1.4	
I don’t know	65	22.5	
7. Paternal support contributes to mothers’ success with breastfeeding.			
True	260	90	90
False	7	2.4	
I don’t know	22	7.6	
8. Breastfeeding strengthens the bond between mother and infant.			
True	272	94.1	94.1
False	4	1.4	
I don’t know	13	4.5	
9. Breast milk supports the infant’s immune system.			
True	278	96.2	96.2
False	2	0.7	
I don’t know	9	3.1	
10. Breastfeeding causes sagging or changes in the shape of the breasts.			
True	120	41.5	22.1
False	64	22.1	
I don’t know	105	36.3	
11. Breastfeeding contributes positively to the mother’s psychological well-being.			
True	258	89.3	89.3
False	2	0.7	
I don’t know	29	10	
12. Mothers should continue breastfeeding their infants during the night.			
True	259	89.6	89.6
False	5	1.7	
I don’t know	25	8.6	
13. If the father does not want the mother to breastfeed, she should not breastfeed her infant.			
True	259	89.6	4.8
False	14	4.8	
I don’t know	16	5.5	
14. Infant formula requires more time and preparation than breastfeeding.			
True	115	39.8	39.8
False	114	39.5	
I don’t know	60	20.8	
15. Every infant has the right to be breastfed.			
True	284	98.3	98.3
False	2	0.7	
I don’t know	3	1	
	Mean ± SD	Med (min–max)	
Number of correct responses	9.96 ± 1.8	10 (2: 15)	

SD: standard deviation; Med: median; Min: minimum; Max: maximum. Note: Response categories with zero frequencies (*n* = 0) are not displayed. For negatively worded statements (Items 3, 4, 10, and 13), “False” was considered the correct response.

**Table 3:** Distribution of participants’ responses to attitude items regarding breast milk and breastfeeding.

	Frequency	Percentage
1. How important do you think it is for a mother to initiate breastfeeding immediately after birth?		
Not important at all	2	0.7
Slightly important	3	1
Moderately important	11	3.8
Very important	273	94.5
2. How important do you think breast milk is for infant growth and development?		
Slightly important	1	0.4
Moderately important	4	1.4
Very important	284	98.3
3. How important do you think the beneficial effects of breastfeeding on maternal health are?		
Not important at all	88	30.5
Not important	3	1
Slightly important	13	4.5
Moderately important	6	2.1
Very important	179	61.9
4. How important do you think it is for you to support your partner with breastfeeding?		
Not important at all	6	2.1
Slightly important	4	1.4
Moderately important	9	3.1
Very important	270	93.4
5. How important do you think antenatal education on breast milk and breastfeeding is for you and your partner for successful breastfeeding?		
Not important at all	42	14.5
Not important	3	1
Slightly important	17	5.9
Moderately important	16	5.5
Very important	211	73

Note: Attitude items were analyzed individually; no total score was calculated. Response categories with zero frequencies (*n* = 0) are not displayed.

**Table 4:** Comparison of the number of correct responses regarding breast milk and breastfeeding according to participants’ sociodemographic and obstetric characteristics.

	Mean ± SD	Med (Min–Max)	Test Statistic	*p*	ES [95% CI]
Educational attainment					
Primary school	10.6 ± 1.52	10 (9: 13)	4.257	0.235 ^x^	0.004 [0.000: 0.047]
Middle school	9.95 ± 2.08	10 (2: 15)			
High school	10.11 ± 1.35	10 (6: 14)			
University	9.63 ± 1.63	10 (6: 13)			
Employment status					
Manual worker	10.01 ± 1.8	10 (3: 15)	0.963	0.810 ^x^	0.000 [0.000: 0.017]
Public-sector employee	10.11 ± 0.99	10 (8: 12)			
Self-employed	9.82 ± 2.01	10 (2: 13)			
Unemployed	9.43 ± 1.72	10 (6: 11)			
Income level					
Income lower than expenses	9.84 ± 2.17	10 (2: 15)	0.866	0.649 ^x^	0.000 [0.000: 0.023]
Income equal to expenses	10.07 ± 1.54	10 (6: 14)			
Income higher than expenses	9.8 ± 1.87	10 (3: 13)			
Pregnancy intendedness					
Unplanned-intended	9.57 ± 1.81	10 (3: 11)	−0.828	0.408 ^y^	0.098 [−0.292: 0.487]
Planned-intended	10 ± 1.8	10 (2: 15)			
Family structure					
Nuclear family	10.01 ± 1.77	10 (3: 15)	−0.272	0.786 ^y^	0.032 [−0.213: 0.277]
Extended family	9.86 ± 1.86	10 (2: 14)			
Mode of delivery					
Vaginal delivery	9.96 ± 1.81	10 (2: 15)	−0.319	0.750 ^y^	0.038 [−0.196: 0.271]
Cesarean delivery	9.96 ± 1.8	10 (3: 14)			
Infant sex					
Female	9.79 ± 1.91	10 (2: 14)	−1.481	0.139 ^y^	0.175 [−0.056: 0.406]
Male	10.12 ± 1.68	10 (3: 15)			
Received education on pregnancy, childbirth, and the postpartum period during the antenatal period					
Yes	10.17 ± 1.95	10 (3: 15)	−2.236	**0.025 ** ^y^	0.265 [0.017: 0.514]
No	9.86 ± 1.72	10 (2: 14)			
Received education on breast milk and breastfeeding during the postpartum period					
Yes	10.09 ± 1.93	10 (2: 15)	−1.870	0.061 ^y^	0.221 [−0.021: 0.464]
No	9.89 ± 1.73	10 (3: 14)			

SD: standard deviation; Med: median; Min: minimum; Max: maximum; ^x^ Kruskal–Wallis H test; ^y^ Mann–Whitney U test. ES [95% CI] = effect size [95% confidence interval]: eta-squared (η^2^H) and equivalent Cohen’s d (d). Bold: Statistically significant results (*p* < 0.05).

**Table 5:** Correlations of participants’ age and their partners’ gestational age with the number of correct responses regarding breast milk and breastfeeding.

	Number of Correct Responses	
	r	*p*
Age	0.013	0.831
Gestational age	0.023	0.702

r: Spearman’s correlation coefficient.

## Data Availability

The data from this study are not publicly available due to ethical considerations regarding participant confidentiality, but they are available from the corresponding author upon reasonable request. Only de-identified excerpts will be provided.

## Supplementary Materials

The following supporting information can be downloaded at https://www.mdpi.com/article/10.3390/healthcare14213803/s1, Table S1. Multivariable robust regression analysis of breastfeeding knowledge scores.
