# Supplementary material for: First-Time Fathers’ Knowledge and Attitudes Toward Breast Milk and Breastfeeding: A Cross-Sectional Study
Source: Healthcare (Basel). 2026 Nov 6;14(21):3803. doi: 10.3390/healthcare14213803 (PMC13762545; doi:10.3390/healthcare14213803)
Supplement: Supplementary file 1 [file healthcare-14-03803-s001.zip › healthcare-4563196-supplementary.pdf]

Supplementary Table S1. Multivariable Robust Regression Analysis of Breastfeeding Knowledge Scores

**Variables**	**B[1] (95% CI)**	**SE**	**β[2]**	**Test statistic**	**p**	**VIF**
Intercept	8.64 (5.673: 11.608)	1.507	---	5.732	**<0.001**	---
Paternal age	0.013 (-0.024: 0.051)	0.019	0.044	0.690	0.491	1.166
Partner's gestational age	0.027 (-0.039: 0.093)	0.034	0.049	0.809	0.419	1.077
**Employment status**						
Manual worker			Reference			
Public sector employee	0.05 (-0.657: 0.757)	0.359	0.009	0.140	0.889	1.187
Self-employed	-0.134 (-0.545: 0.276)	0.209	-0.039	-0.644	0.520	1.070
Unemployed	-0.318 (-1.418: 0.783)	0.559	-0.034	-0.568	0.570	1.059
**Income level**						
Income less than expenses			Reference			
Income equal to expenses	0.027 (-0.358: 0.412)	0.196	0.009	0.137	0.891	1.338
Income greater than expenses	-0.16 (-0.725: 0.406)	0.287	-0.040	-0.556	0.579	1.481
**Pregnancy planning status**						
Unplanned but wanted			Reference			
Planned and wanted	0.32 (-0.234: 0.875)	0.282	0.068	1.137	0.256	1.025
**Family structure**						
Nuclear family			Reference			
Extended family	-0.019 (-0.384: 0.345)	0.185	-0.006	-0.105	0.916	1.080
**Mode of delivery**						
Vaginal delivery			Reference			
Cesarean delivery	0.004 (-0.349: 0.358)	0.179	0.002	0.024	0.981	1.103
**Infant sex**						
Female			Reference			
Male	0.25 (-0.085: 0.585)	0.170	0.088	1.470	0.143	1.021
**Antenatal education on pregnancy, childbirth, and the postpartum period**						
Yes			Reference			
No	-0.377 (-0.839: 0.086)	0.235	-0.123	-1.604	0.110	1.686
**Postpartum education on breast milk and breastfeeding**						
Yes			Reference			
No	-0.133 (-0.586: 0.321)	0.230	-0.044	-0.577	0.565	1.707

F = 1.042; p = 0.412; R^2 = 4.69%; [1] Unstandardized regression coefficient; [2] Standardized regression coefficient; 95% CI: Bootstrap confidence interval; VIF: Variance Inflation Factor.
